# Cognitive and affective interaction with somatosensory afference in acupuncture–a specific brain response to compound stimulus

**DOI:** 10.3389/fnhum.2023.1105703

**Published:** 2023-06-21

**Authors:** Changjin Jung, Jieun Kim, Kyungmo Park

**Affiliations:** ^1^Department of Electronics and Information Convergence Engineering, Kyung Hee University, Yongin, Republic of Korea; ^2^Division of KM Science Research, Korea Institute of Oriental Medicine, Daejeon, Republic of Korea; ^3^Department of Biomedical Engineering, Kyung Hee University, Yongin, Republic of Korea

**Keywords:** brain interaction, somatosensory afference, interoceptive attention, acupuncture, therapeutic contextual manipulation

## Abstract

**Introduction:**

Acupuncture is a clinical intervention consisting of multiple stimulus components, including somatosensory stimulation and manipulation of therapeutic context. Existing findings in neuroscience consolidated cognitive modulation to somatosensory afferent process, which could differ from placebo mechanism in brain. Here, we aimed to identify intrinsic process of brain interactions induced by compound stimulus of acupuncture treatment.

**Methods:**

To separately and comprehensively investigate somatosensory afferent and cognitive/affective processes in brain, we implemented a novel experimental protocol of contextual manipulation with somatosensory stimulation (real acupuncture: REAL) and only contextual manipulation (phantom acupuncture: PHNT) for fMRI scan, and conducted independent component (IC)-wise assessment with the concatenated fMRI data.

**Results:**

By our double (experimentally and analytically) dissociation, two ICs (CA1: executive control, CA2: goal-directed sensory process) for cognitive/affective modulation (associated with both REAL and PHNT) and other two ICs (SA1: interoceptive attention and motor-reaction, SA2: somatosensory representation) for somatosensory afference (associated with only REAL) were identified. Moreover, coupling between SA1 and SA2 was associated with a decreased heart rate during stimulation, whereas CA1 was associated with a delayed heart rate decrease post-stimulation. Furthermore, partial correlation network for these components demonstrated a bi-directional interaction between CA1 and SA1/SA2, suggesting the cognitive modulation to somatosensory process. The expectation for the treatment negatively affected CA1 but positively affected SA1 in REAL, whereas the expectation positively affected CA1 in PHNT.

**Discussion:**

These specific cognitive-somatosensory interaction in REAL were differed from vicarious sensation mechanism in PHNT; and might be associated with a characteristic of acupuncture, which induces voluntary attention for interoception. Our findings on brain interactions in acupuncture treatment elucidated the underlying brain mechanisms for compound stimulus of somatosensory afferent and therapeutic contextual manipulation, which might be a specific response to acupuncture.

## 1. Introduction

Acupuncture is a multi-dimensional intervention. Needle stimulation, the essential component of the acupuncture procedure, is known to induce different types of somatosensory afferent activation and consequent modulatory effects on the brain (Chae et al., 2013; Maeda et al., 2017; Kim et al., 2020), as well as responses in other systems, including the immune and endocrine systems (Wei and Hsieh, 2020; Liu et al., 2021; Oh and Kim, 2021). Concurrently, in clinical settings, the needling procedure is necessarily accompanied by additional and supplementary procedures (e.g., preparations of needle insertion), which may potentially lead to various therapeutic contexts. The manipulation of therapeutic conditions in acupuncture (Cao et al., 2018) suggests that not only somatosensory but also other sensory modalities and related cognition are involved in acupuncture in the brain.

Indeed, existing findings in neuroscience consolidated cognitive modulation to somatosensory process in brain. Primary somatosensory cortex (SI) was modulated by attentional process (Steinmetz et al., 2000; Bardouille et al., 2010; Dockstader et al., 2010), cognitive imagery (Jafari et al., 2020), and action understanding (Valchev et al., 2017). Moreover, an animal study showed that decision and reward history affected the neuronal activity in the primary sensory thalamus in the ascending sensory pathway (Waiblinger et al., 2018). These brain mechanism suggests that somatosensory afference and contextual manipulation of acupuncture, a compound stimulus, can be interacted in brain, which differs from placebo mechanism in brain (Wager and Atlas, 2015).

In our previous studies, we implemented an experimental condition of sham acupuncture (phantom acupuncture, PHNT), which dissociated somatosensory afferents of needling stimulation from the total effect of acupuncture treatment (Lee et al., 2014, 2019; Makary et al., 2018). PHNT induced needling credibility using only contextual manipulation of visual information in the stimulation session with verbal instruction in the preparatory session, and showed activation of multiple brain regions (e.g., dorsolateral prefrontal cortex and ventrolateral prefrontal cortex). These brain regions could be associated with the cognitive/affective process (Makary et al., 2018) and potentially lead to an expectation-related placebo analgesic effect (Lee et al., 2019). Moreover, actual needling stimulation in addition to the same protocol of real acupuncture (REAL) showed greater activation in brain regions associated with somatosensory afferents (e.g., the posterior insular cortex and somatosensory cortex) than PHNT. Nevertheless, the model-based voxel-wise assessment of functional brain data in previous studies had limitations for taking account of underlying brain intrinsic processes.

In fact, REAL and PHNT induced not only therapeutic components of somatosensory afferent or cognitive/affective but also non-therapeutic components, which were implicitly engaged with the protocols. Moreover, potential therapeutic components could exist in both REAL and PHNT and may interact with other components. For instance, while both REAL and PHNT induced cognitive/affective brain regions as expected, their associations with expectations for acupuncture treatment differed between REAL and PHNT. Furthermore, repeated needling stimulations in a session showed heterogeneous brain response patterns (Napadow et al., 2013), indicating intrinsic interactions around somatosensory afferent process in the brain. These phenomena can be considered in the typical setting of acupuncture and its related therapeutic outcomes.

Here, we conducted another REAL/PHNT experiment with twenty-five healthy participants for fMRI scan, and independent component (IC)-wise assessment with the concatenated fMRI data to investigate underlying brain intrinsic processes induced by acupuncture. Independent component analysis (ICA) separated brain responses to contrasted experimental paradigms into ICs (i.e., data-driven approach) and generalized linear model (GLM) assesses their significances for experimental protocols (i.e., model-based approach). By combining experimental dissociation (i.e., REAL and PHNT) and analytic dissociation (i.e., IC-wise GLM), the intrinsic brain components were identified. Moreover, we assessed the psychological (i.e., somatosensory sensation) and physiological [i.e., heart rate (HR)] measures of REAL and PHNT to elucidate the functional characteristics of the brain components.

## 2. Materials and methods

### 2.1. Experimental design

We designed an experimental condition to remove the somatosensory afferent from the acupuncture treatment (PHNT). PHNT led to visual afferent and needling credibility without somatosensory afferents by displaying a video for needling stimulation with verbal instruction inducing a context of acupuncture treatment. In contrast to PHNT, REAL led to somatosensory afferents by conducting needling stimulation in addition to PHNT. The experimental designs for REAL and PHNT were identical to those used in previous studies (Lee et al., 2014; Makary et al., 2018). The participants were randomly and evenly assigned to the crossover design of REAL-PHNT or PHNT-REAL. By concatenating the time series of BOLD for REAL and PHNT sessions, ICA could separate out different brain patterns induced by acupuncture treatment.

#### 2.1.1. REAL session

A magnetic resonance imaging (MRI)-compatible needle (0.3 mm x 30 mm, titanium needle, DongBang Co., Korea) was inserted into the left ST36 (Zusanli) before the MRI scan started (Supplementary Figure 1). During the 420 s functional MRI (fMRI) scan, a licensed acupuncturist manually performed 24 or 22 needle stimulations by rotating the needle at approximately 1 Hz for 2 s (inter-stimulation interval between stimulations: 17.0 ± 2.2 s for the 24 stimulations; 19.0 ± 2.9 s for the 22 stimulations). Since the acupuncturist’s hand approaching to needle for stimulations might induce the participant’s anticipation of stimulation before the actual stimulations were delivered, the timings of the hand approach were also randomized (inter-stimulation interval between the hand approach and the stimulation: 5.9 ± 1.7 s for the 24 stimulations; 6.0 ± 1.6 s for the 22 stimulations). A camera in the MRI room recorded the repeated manipulation of the acupuncture needle on the left ST36 with randomized inter-stimulus interval and simultaneously displayed it to the participants during the scan (Supplementary Figure 1). The participants watched the video stream for the needle stimulation at their left leg, which was visual information for the therapeutic context (i.e., contextual manipulation). The recordings were used in the PHNT sessions of the participants and other participants. The needle was removed after the completion of the scan.

#### 2.1.2. PHNT session

PHNT was performed identically to REAL from the participant’s point of view, except that the needle was not inserted into the acupoint (ST36). To ensure participants’ needling credibility without needling insertion, we played the recorded video clip from the participant’s REAL session or the best-matched participant’s REAL session (i.e., contextual manipulation). The acupuncturist mimicked the acupuncture procedures of needle insertion and rotation in synchronizing with the acupuncturist’s action in the video clip during the PHNT session (Supplementary Figure 1).

### 2.2. Participants

Participants were recruited *via* fliers/webpages and screened for the following exclusion criteria: (1) age below 19 years; (2) mental or respiratory syndrome, claustrophobia, depression, bipolar disorder, anorexia nervosa, or a generalized psychiatric history within the past year; (3) poor eyesight with uncorrected vision; and (4) respiratory syndrome due to pulmonary vascular disease and physical causes. Participants were asked to not take any medication or caffeine before the MRI scan. Twenty-five healthy, right-handed adults (16 men, 13 women; 27.8 ± 3.4 years old) without previous experience of acupuncture were enrolled in this study. All the participants provided informed consent. The study procedures were approved by the Institutional Review Board of Kyung Hee University Hospital in Gangdong (approval number: KHNMC-OH-IRB 2009-006).

Needling credibility for PHNT was assessed by structural interviews at the end of the experiment, and only 19 participants (12 men, 7 women; 10 subjects for the REAL-PHNT order, 9 subjects for the PHNT-REAL order) who reported needling credibility were included in the analysis.

### 2.3. Brain image acquisition and preprocessing

Brain images were acquired using a 3T Philips MRI System (Philips, Achieva, Best, Netherlands) with an 8-channel head coil. Whole brain BOLD images were acquired using a T2*-weighted echo-planar sequence (TR/TE = 2000/30 ms, flip angle = 90°, voxel size = 2.9 × 2.9 × 4.0 mm^3^, axial slice number = 34; matrix = 64 × 64) for REAL and PHNT scans (420 s).

Preprocessing of the BOLD images was performed using analysis packages of FSL (FMRIB Software Library, Oxford, UK) and FreeSurfer (Martinos Center for Biomedical Imaging, Boston, MA, USA). Physiological noise was corrected by RETROICOR (Glover et al., 2000) with the timing of the QRS peak and respirational phase information. Head motion was corrected with an affine transformation procedure using FSL-MCFLIRT (Jenkinson et al., 2002), and the skull region was extracted using FSL-BET (Smith, 2002). Cortical surface of T1-weighted images (TR/TE = 2.73/3.19 ms, flip angle = 7°, FOV = 256 × 256 mm^2^; slice thickness = 1.33 mm) was reconstructed using FreeSurfer’s recon-all (Dale et al., 1999), and co-registration between anatomical and functional images was completed using FreeSurfer’s register tool (Greve and Fischl, 2009). Images in the native space were spatially normalized to the Montreal Neurological Institute (MNI) space using FSL-FNIRT, and spatial smoothing with a 5 mm full width at half maximum Gaussian kernel was completed. Then, temporal high-pass filtering was performed with a 0.015-Hz cut-off frequency. Following preprocessing, we excluded two participants due to excessive head movement (over 2 mm displacement between successive image volumes) and two participants due to distortion of raw BOLD data with scanner issues. Fifteen pairs of REAL and PHNT scans were used for further analyses.

### 2.4. Brain response assessment using an IC-wise generalized linear model analysis

**FIGURE 1:**
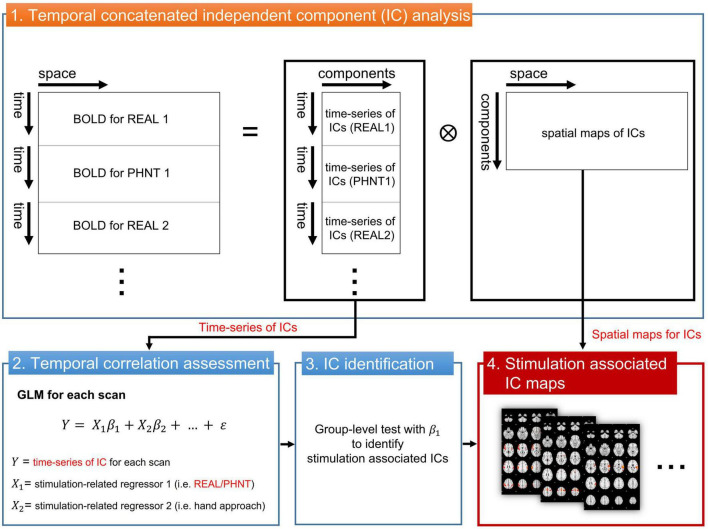
IC-wise generalized linear model (IC-wise GLM) analysis. The temporal concatenated ICA decomposed time-series of whole brain voxels into pairs of time-series and spatial maps for each IC. Then, GLM analysis was performed for time-series of each IC (dependent variable) with multiple stimulation-related regressors, including REAL/PHNT and anticipation (hand approach). Finally, protocol-dependent ICs were identified in a group-level test with *t*-statistics of β_1_. The significance level was corrected using a Bonferroni correction for multiple comparisons (the number of ICs). IC, independent component; GLM, generalized linear model; ICA, IC analysis.

To assess brain correlates for REAL and PHNT responses, the independent component (IC) time-series instead of the voxel-wise fMRI time series was extracted and used to fit with a GLM regressor from our repeated stimulation-related design (Figure 1). The temporal concatenated ICA decomposed the whole-brain BOLD image into pairs of common spatial maps and their associated time-series for each IC. BOLD images from 15 pairs of REAL and PHNT scans were concatenated along the time series, and FSL_MELODIC completed ICA with full-temporal concatenation (Beckmann and Smith, 2004). The number of ICs was estimated using Bayesian dimensionality estimation techniques (Beckmann and Smith, 2004). The ICA componentized the BOLD activations into ICs, which was much less than the number of whole brain voxels. GLM analysis with multiple event-related regressors, including the stimulation paradigm of the REAL/PHNT, was performed to assess associations between ICs and acupuncture treatment using the R Statistical Package (R-Core-Team, 2012). Time-series of ICs were applied as dependent variables of GLM instead of BOLD images for conventional GLM analysis, and two regressors of the stimulation and the hand approach were used as explanatory variables in the GLM analysis. The regressors were independently generated by convolution with a canonical double gamma hemodynamic response function (HRF, FSL-FEAT), and GLM analysis carried out parameter estimates (PEs) of the stimulus and hand approach for each IC. To define the best-fit IC to REAL/PHNT protocols, we tested the significance of the *t*-statistics of PEs for REAL and PHNT using a one-sample *t*-test. The significance level was corrected using Bonferroni correction for multiple comparisons of the number of ICs. Thus, the IC-wise GLM assessed significantly correlated ICs with the stimulation protocols of REAL/PHNT.

Moreover, we assessed ICs associated with a single 2-s stimulation using one-shot regressors corresponding to each stimulation of the 24 or 22 repeated stimuli in the GLM analysis. GLM analysis with the one-shot stimulation regressor and hand approach regressor carried out PEs for each stimulation. Outliers for the PEs were tested using Grubbs’ test at a significance level of α < 0.05 and were excluded from the concordance analysis.

Additionally, we assessed the temporal characteristics of IC responses using data-driven optimization of the HRF for REAL. The FMRIB’s linear optimal basis sets (Woolrich et al., 2004) were used for GLM instead of canonical HRF convolution. Combinations of PEs for the three basis functions yielded the optimized HRFs for IC responses.

### 2.5. Brain response assessment using voxel-wise GLM

Voxel-wise GLM analysis was conducted for whole brain data and compared with the IC-wise GLM. The multiple regressors for event-related REAL and the hand approach were independently generated by convolution with HRF. In addition, six translation/rotation motion parameters from FSL-MCFLIRT and motion-outlier confound matrices from FSL_motion-outliers were added to the GLM as nuisance regressors. The resulting PEs for REAL were passed up to group-level GLM analysis (FSL-FEAT, mixed-effect model) for one-sample *t*-test. Whole brain maps from the group-level analysis were threshold at *z* > 3.29 and cluster corrected for multiple comparisons (FWE, *P* < 0.01) (Figure 3A).

To validate the threshold of the spatial maps for IC-wise GLM, the optimized threshold value corresponding to the maximum spatial cross-correlation coefficient between the covering area for threshold spatial maps from IC-wise GLM and the group average map from voxel-wise GLM for REAL was evaluated (Figure 3B). We found that the threshold for the maximum spatial cross-correlation coefficient was at *z* = 4.5 (*R* = 0.44) and the spatial maps for IC-wise GLM were thresholded at *z* = 4.5.

### 2.6. Heart rate response assessment

Electrocardiogram (ECG) signals were acquired using a PowerLab system (PowerLab 1200, ADInstruments, Australia) with MR-compatible Ag/Ag-Cl electrodes during the REAL/PHNT scans. HR was calculated using ECG QRS peak annotation and resampled to 1 Hz. The average HR was calculated for the 2-s REAL/PHNT compared to the 2-s baseline prior to each stimulation. Since HRs showed delayed changes to stimulation, the average HR for post-stimulation was calculated for 4 s after stimulation compared to the same 2-s baseline. HR changes were averaged for REAL/PHNT.

### 2.7. Behavioral assessments

Before the fMRI scans, participants were asked to complete acupuncture efficacy expectation questionnaires to assess a belief in treatment expectation for acupuncture (Dennehy et al., 2002). The questionnaire was scored using a five-point Likert-type scale, and the average score of the “general belief” items was used for the expectation assessment. After the fMRI scans, somatosensory sensations for REAL/PHNT were assessed using the Massachusetts General Hospital Acupuncture Sensation Scale (MASS) index (Kong et al., 2007).

### 2.8. Statistical analysis

All statistical analyses were performed using the R Statistical Package, and all data were first tested for a normal distribution using the Shapiro–Wilk test. A one-sample *t*-test or the Wilcoxon signed-rank test was used to evaluate the IC-wise brain response, HR changes, and somatosensory sensations for REAL/PHNT. Paired differences in IC-wise brain responses, behavioral measures, and HR responses between the REAL and PHNT groups were assessed using the paired two-sample *t*-test or Wilcoxon signed-rank test. *P*-values for IC-wise GLM were corrected using a Bonferroni correction for multiple comparisons (number of ICs). Associations between IC-wise brain responses, behavioral measures, and HR responses were assessed using Pearson’s or Spearman’s correlation analysis. Concordance correlation coefficients (Lin, 1989) were calculated to assess the concordance between IC response patterns and the repeated stimuli of REAL/PHNT. The resulting PEs of the one-shot stimulation regressors in the IC-wise GLM were normalized within each scan, and concordance was assessed between two different ICs using pairs of normalized PEs. A repeated-measures analyses of variance (ANOVAs) and paired *t*-tests (*post hoc*) were used to assess differences in the peak timings of the optimized HRF between ICs. To assess relationship among multiple variables, Spearman’s partial correlation coefficients between paired of variables were computed by controlling other variables. The significance level for all statistical tests was set at α = 0.05.

## 3. Results

### 3.1. Somatosensory sensation induced by REAL and PHNT

Contextual manipulation without needling stimulation in PHNT induced somatosensory credibility in 76% of participants (19 of 25 participants). Our acupuncture protocols significantly induced a somatosensory sensation (MASS index scores) in both REAL (4.32 ± 2.43, *p* < 0.01) and PHNT (1.23 ± 1.18, *p* < 0.01) with somatosensory credibility (Figure 4B). REAL induced a significantly greater somatosensory sensation than PHNT (*p* < 0.01).

### 3.2. Heart rate changes induced by REAL and PHNT

REAL induced significant HR decreases during 2-s stimulations compared to the baseline before stimulation (*p* = 0.007) (Figure 4B). Moreover, REAL induced significant HR decreases during 4 s of post-stimulations compared to the baseline (*p* = 0.010). There was no significant HR decreases during 2-s stimulations in PHNT. However, PHNT induced significant HR decreased during 4 s after stimulations (*p* = 0.024) (Figure 4B). REAL showed greater HR decreases than PHNT for both 2-s stimulations and 4 s of post-stimulations (*P* < 0.05) (Figure 4B).

### 3.3. Brain responses to REAL/PHNT: IC-wise GLM analyses

**FIGURE 2:**
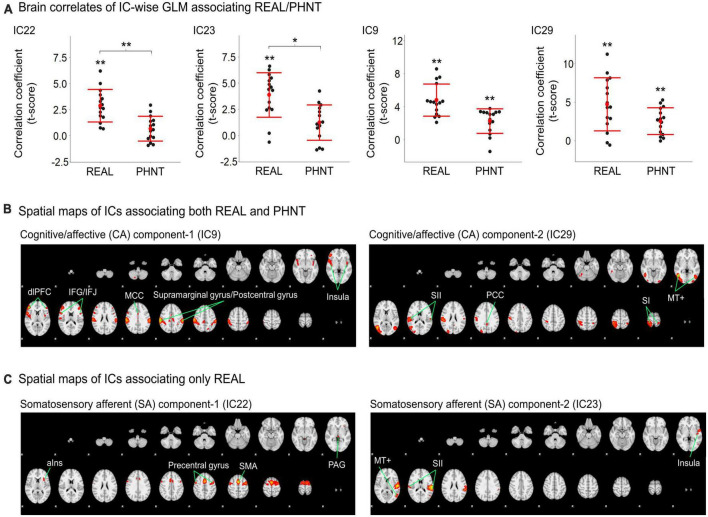
Significant independent components (ICs) for REAL and PHNT assessed by IC-wise GLM. **(A)** IC9 (*V* = 120, *p* < 0.01), IC22 (*t* = 2.87, *p* < 0.001), IC23 (*t* = 3.86, *p* < 0.001), and IC29 (*t* = 4.72, *p* = 0.002) showed significant positive correlation with the stimulation protocol for REAL. IC9 (*V* = 116, *p* = 0.01) and IC29 (*t* = 2.52, *p* = 0.001) showed significant positive correlations with the stimulation protocol for PHNT. Correlates of IC22 and IC23 were significantly stronger in REAL than PHNT (IC23: *p* = 0.008; IC23: *p* = 0.025). *P*-values were corrected using a Bonferroni correction for multiple comparisons; *p < 0.05, **p < 0.01; **(B)** Spatial maps for the significant ICs. SA1 for IC22 includes the left anterior insula cortex (aIns), supplementary motor area (SMA), precentral gyrus, and periaqueductal gray (PAG); and SA2 for IC23 includes the secondary somatosensory cortex (SII), posterior insula (pIns), and V5/MT+; **(C)** CA1 for IC9 includes the insular cortex, dorsolateral prefrontal cortex (dlPFC), inferior frontal gyrus (IFG), middle cingulate cortex (MCC), postcentral gyrus, and supramarginal gyrus; and CA2 for IC29 includes V5/MT+, the primary somatosensory cortex (SI), secondary somatosensory cortex (SII), parietal lobe (PL), and posterior cingulate cortex (PCC). The spatial maps showed a threshold at *z* = 4.50, which showed the maximum spatial cross-correlation coefficient with the group average map from the voxel-wise GLM for REAL (threshold at *z* > 3.29; FWE with *P* < 0.01).

Temporal concatenation with ICA decomposed the 39 ICs based on Bayesian dimensionality estimation. Across the 39 ICs, we found that four ICs correlated with REAL and two ICs correlated with PHNT using a one-sample *t*-test with a Bonferroni correction for multiple comparisons (Figure 2A). In REAL, IC9 (*V* = 120, *p* < 0.01, Wilcoxon signed-rank test), IC22 (*t* = 2.87, *p* < 0.001), IC23 (*t* = 3.86, *p* < 0.001), and IC29 (*t* = 4.72, *p* = 0.002) showed significant positive correlations with the stimulation protocol. In PHNT, IC9 (*V* = 116, *p* = 0.01, Wilcoxon signed rank test) and IC29 (*t* = 2.52, *p* = 0.001) showed significant positive correlations with the stimulation protocol. Moreover, IC22 and IC23 showed a significantly greater correlation with the stimulation protocol in REAL than with PHNT (IC23: *p* = 0.008; IC23: *p* = 0.025), indicating that IC22 and IC23 originated from somatosensory afferents. The correlation with the stimulation protocol did not differ between the REAL and PHNT for IC9 (*p* = 0.08) and IC29 (*p* = 0.64). Thus, we identified IC9 and IC29 as cognitive/affective process (CA) components (CA1 = IC9, CA2 = IC29), and IC22 and IC23 as somatosensory afferent (SA) components (SA1 = IC22, SA2 = IC23). We confirmed that there was no learning effect of REAL to the brain response of PHNT by assessing differences of coefficients of these ICs between participants with REAL-first and PHNT-first orders (two-sample *t*-test; IC9: *p* = 0.299, IC22: *p* = 0.855, IC23: *p* = 0.223 IC29: *p* = 0.148).

**FIGURE 3:**
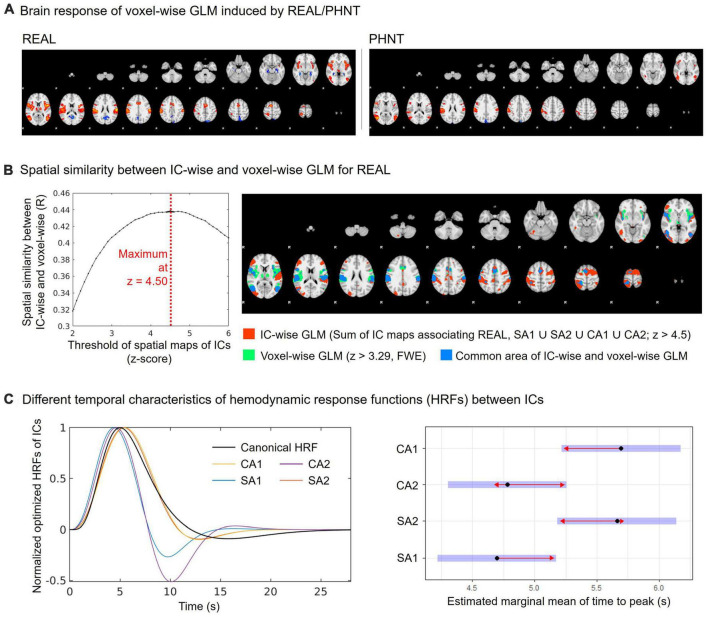
Spatial and temporal characteristics of CA and SA components. **(A)** Group average map of the brain response by REAL using voxel-wise GLM (threshold at *z* > 3.29; FWE with *P* < 0.01); **(B)** Spatial similarity between IC-wise and voxel-wise GLM for REAL. The IC-wise map for REAL included the special maps for SA1, SA2, CA1, and CA2. The IC-wise map for REAL showed the maximum spatial cross-correlation coefficient with group average map from the voxel-wise GLM for REAL at threshold of *z* = 4.50 (*R* = 0.44); **(C)** Repeated-measures ANOVA showed different peak-timings of optimized HRF between ICs (CA1: 4.70 ± 0.63 s, CA2: 5.66 ± 1.04 s, SA1: 5.70 ± 1.06 s, SA2: 4.78 ± 0.32 s; *F* = 5.22, *p* < 0.01). The peak-timings of CA1 were later than SA1 (Tukey Test, *p* = 0.03), and the peak-timings of SA2 were later than SA1 (Tukey Test, *p* = 0.03).

**FIGURE 4:**
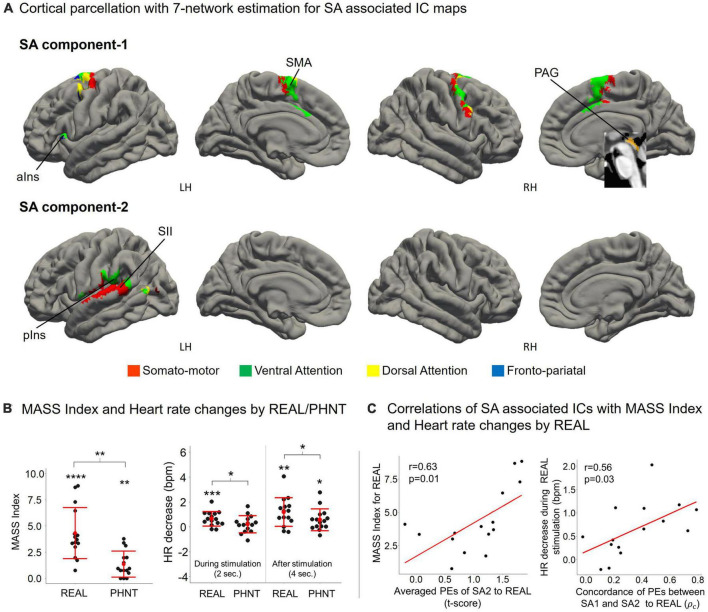
**(A)** SA components include the overall somato-motor and ventral attention networks. ICA separated somato-motor and ventral attention networks into medial subregions for SA1 and lateral subregions for SA2. SA1 includes the anterior insular cortex (aIns), supplementary motor area (SMA), and periaqueductal gray (PAG); and SA2 includes the secondary somatosensory cortex (SII) and posterior insula (pIns); **(B)** MASS index scores for the acupuncture sensation were significant for both REAL (4.32 ± 2.43, *p* < 0.01) and PHNT (1.23 ± 1.18, *p* < 0.01). REAL induced a significantly stronger somatosensory sensation than PHNT (*p* < 0.01). REAL induced significant heart rate decreases during 2-s stimulations (*p* < 0.01). Both REAL and PHNT induced significant heart rate decreases during the 4 s after stimulation (*p* < 0.01 for REAL, *p* < 0.05 for PHNT). REAL induced a stronger heart rate decrease than PHNT during both 2-s stimulations and 4 s after stimulation (*P* < 0.05). *p < 0.05, **p < 0.01, ***p < 0.005, and ****p < 0.001. **(C)** Mass index score was positively correlated with the averaged PEs of SA2 (*r* = 0.63, *p* = 0.01). Heart rate decreases during REAL stimulation were positively correlated with the concordance of PEs between SA1 and SA2 (*r* = 0.56, *p* = 0.03). ρ_*c*_: concordance correlation coefficient.

Spatial maps with the optimized threshold (*z* = 4.5) demonstrated the brain regions associated with the identified ICs (Table 1 and Figures 2B, C). CA1 includes the insular cortex, dorsolateral prefrontal cortex (dlPFC), inferior frontal gyrus (IFG), middle cingulate cortex (MCC), postcentral gyrus, and supramarginal gyrus; CA2 includes V5/MT+, SI, secondary somatosensory cortex (SII), parietal lobe (PL), and posterior cingulate cortex (PCC) (Figure 2B). SA1 includes the left anterior insular cortex (aIns), supplementary motor area (SMA), precentral gyrus, and periaqueductal gray (PAG), and SA2 includes the secondary somatosensory cortex (SII), posterior insular (pIns), and V5/MT+ (Figure 2C).

**TABLE 1:** Spatial maps of ICs associating somatosensory afferent (SA1 and SA2) and cognitive/affective (CA1 and CA2) process.

ICs	Brain regions	Voxels	Peak voxels (MNI)			*Z*-score^a^
			x	y	z	
IC9 (CA1)	Right supramarginal gyrus	2902	62	−26	40	16.08
	Right dorsolateral prefrontal cortex	2483	46	40	8	12.72
	Left inferior parietal lobule	1522	−58	−28	44	12.29
	Left middle insula cortex	354	−38	−2	12	7.09
	Left dorsolateral prefrontal cortex	348	−46	38	16	7.91
	Left middle cingulate cortex	333	−8	−24	40	7.03
	Right middle cingulate cortex	225	6	−32	46	8.12
	Left inferior frontal gyrus	93	−52	8	16	6.21
	Left superior parietal lobule	59	−16	−70	50	5.93
	Right cerebellum (VIII)	51	18	−74	−50	6.10
	Left ventromedial prefrontal cortex	34	−32	38	−10	6.21
	Left cerebellum (VII)	21	−16	−76	−52	5.09
	Right superior parietal lobule	17	22	−66	52	5.13
	Right ventromedial prefrontal cortex	15	28	34	−10	5.11
	Middle cingulate cortex	12	0	−30	30	4.79
IC22 (SA1)	Supplementary motor area	4485	0	4	56	12.89
	Left anterior insula cortex	134	−32	16	8	6.96
	Periaqueductal gray	74	0	−30	−2	6.20
	Right anterior insula cortex	23	34	24	4	5.28
IC23 (SA2)	Left secondary somatosensory cortex/Posterior insula cortex	2333	−56	−16	10	8.96
	Left MT+	264	−50	−68	8	5.95
	Right secondary somatosensory cortex	115	68	−18	16	5.89
IC29 (CA2)	Right MT+	2669	50	−66	2	15.20
	Right primary somatosensory cortex/Parietal lobule	1900	40	−40	62	10.33
	Left MT+	697	−46	−72	4	9.43
	Left primary somatosensory cortex/Parietal lobule	251	−38	−44	62	6.43
	Left visual area 3	212	−30	−94	−2	6.21
	Right cerebellum (Crus I)	142	40	−66	−26	5.59
	Left secondary somatosensory cortex	142	−58	−20	20	6.61
	Posterior cingulate cortex	109	−2	−48	32	5.44
	Right inferior temporal gyrus	53	46	−44	−14	6.39
	Left superior frontal gyrus	22	−16	38	42	5.14

Spatial maps were determined by IC-wise GLMx with a threshold at z < 4.50 and cluster > 10 voxels; ^a^Z-scores represent unnormalized z-statistics of peak voxels from the temporal concatenated ICA. IC, independent component; ICA, IC analysis; GLM, general linear model.

### 3.4. Spatial and temporal characteristics of CA and SA components

The spatial similarity of brain responses between IC-wise and voxel-wise GLMs (Figure 3A) is shown in Figure 3B. Conjunction map of the thresholded (threshold *z* = 4.50) spatial maps of the identified ICs showed the maximum spatial cross-correlation coefficient with the group map from the voxel-wise GLM for REAL (*R* = 0.44) (Figure 3B).

In addition, we found different temporal characteristics of the ICs (Figure 3C). Repeated-measures ANOVA showed different peak-timings of optimized HRF between ICs (CA1: 4.70 ± 0.63 s, CA2: 5.66 ± 1.04 s, SA1: 5.70 ± 1.06 s, SA2: 4.78 ± 0.32 s; *F* = 5.22, *p* < 0.01). The peak timings of CA1 were later than those of SA1 (Tukey test, *p* = 0.03), and the peak timings of SA2 were later than those of SA1 (Tukey test, *p* = 0.03).

### 3.5. Functional characteristics of SA components

Cortical parcellation with 7-network estimation by intrinsic functional connectivity (Yeo et al., 2011) demonstrated that somatosensory-motor and ventral attention networks are composed of SA components (Figure 4A). ICA separated somatosensory-motor and ventral attention networks into medial subregions for SA1 and lateral subregions for SA2.

The somatosensory afferent (needling stimulation) of REAL induced a significant MASS index score for somatosensory sensation (Figure 4B), which was positively correlated with the average PEs of SA2 (*r* = 0.63, *p* = 0.01) (Figure 4C). Thus, greater activation of SA2 induces higher somatosensory sensations. Moreover, REAL induced significant HR decreases during both 2-s stimulations and 4 s of post-stimulations (Figure 4B) compared to the baseline before stimulation. The HR decreased during REAL stimulation and was positively correlated with the concordance of PEs between SA1 and SA2 (*r* = 0.56, *p* = 0.03) (Figure 4C), indicating that more synchronized activation of SA1 and SA2 was associated with greater HR decreases during stimulation.

### 3.6. Functional characteristics of CA components

**FIGURE 5:**
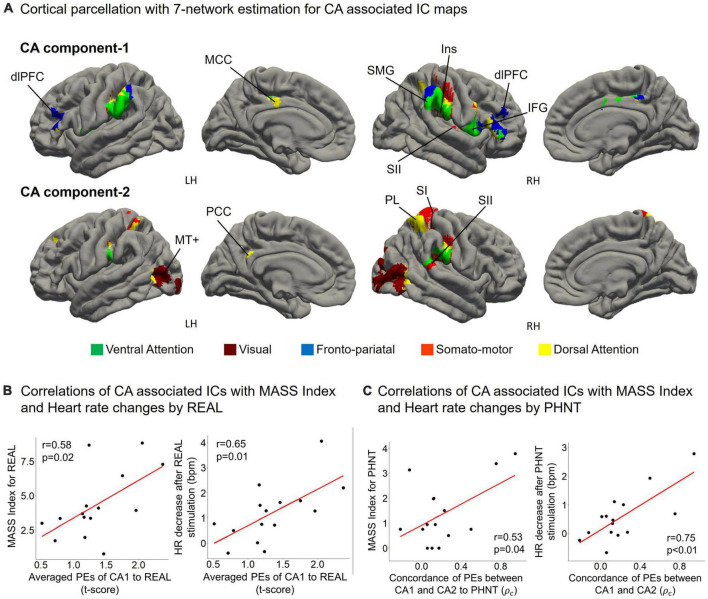
**(A)** CA1 mainly belongs to the fronto-pariatal network, whereas CA2 includes the dorsal attention, visual, and somato-motor networks. CA1 includes the insular cortex (Ins), dorsolateral prefrontal cortex (dlPFC), inferior frontal gyrus (IFG), middle cingulate cortex (MCC), and supramarginal gyrus (SMG); and CA2 includes V5/MT+, the primary somatosensory cortex (SI), secondary somatosensory cortex (SII), parietal lobe (PL), and posterior cingulate cortex (PCC); **(B)** Averaged PEs of CA1 were positively correlated with MASS index scores for acupuncture sensation in REAL (*r* = 0.58, *p* = 0.02) and the heart rate decreased during the 4 s after REAL stimulation (*r* = 0.65, *p* = 0.01); **(C)** MASS index scores for PHNT were positively correlated with the concordance of PEs between CA1 and CA2 (*r* = 0.53, *p* = 0.04). Heart rate decreased during the 4 s after stimulation and was positively correlated with the concordance of PEs between CA1 and CA2 in the PHNT. ρ_*c*_: concordance correlation coefficient.

Cortical parcellation estimation showed that CA1 mainly belongs to the fronto-pariatal network, whereas CA2 belongs to the dorsal attention, visual, and somato-motor networks (Figure 5A). Averaged PEs of CA1 were positively correlated with MASS index scores for REAL (*r* = 0.58, *p* = 0.02), and the HR decreased during the 4 s after REAL stimulation (*r* = 0.65, *p* = 0.01) (Figure 5B). Thus, somatosensory sensations induced by REAL are associated with both SA2 and CA1.

On the other hand, PHNT also induced significant MASS index score (*p* < 0.01), which was significantly lower than that of REAL (*p* < 0.01) (Figure 4B). MASS index scores for PHNT were positively correlated with the concordance of PEs between CA1 and CA2 (*r* = 0.53, *p* = 0.04) (Figure 5C), indicating that a more synchronized activation of CA1 and CA2 was associated with higher somatosensory sensations. Moreover, the PHNT-induced HR decreased 4 s after stimulation (*p* < 0.05) (Figure 4B), which was positively correlated with the concordance of PEs between CA1 and CA2 to PHNT (*r* = 0.75, *r* < 0.01) (Figure 5C). The decrease in HR after PHNT stimulation was significantly lower than that after REAL (*p* < 0.05) (Figure 4B). There was no decrease in HR during PHNT stimulation (*p* = 0.25).

### 3.7. Relationship between SA/CA components and expectation for acupuncture treatment

**FIGURE 6:**
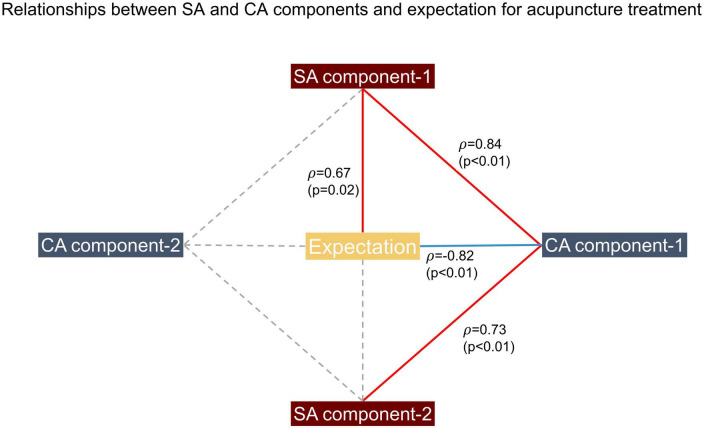
Partial correlation network for SA/CA components and expectation for acupuncture treatment. Spearman’s partial correlation analysis was performed with the four components and expectation scores. Pretrial correlation coefficients for pairs of variables were computed by controlling other variables. CA1 showed significant positive correlation with SA1 (ρ = 0.84, *p* < 0.01) and SA2 (ρ = 0.73, *p* = 0.04). Expectation scores in REAL showed significant positive correlation with SA1 (ρ = 0.67, *p* = 0.02), and inverse correlation with CA1 (ρ = –0.82, *p* < 0.01).

Partial correlation network for SA and CA components showed component-to-component relationships in REAL (Figure 6). Partial correlation analysis performed for two SA and two CA components. Partial correlation coefficients were computed between pairs of components, while controlling for the effect of the remaining two components. CA1 showed positive correlation with SA1 (ρ = 0.69, *p* < 0.01) and SA2 (ρ = 0.58, *p* = 0.04) by controlling other two components (Table 2). Moreover, we included the expectation scores for the treatment in the partial correlation analysis to assess direct effects of the expectation on SA/CA components in REAL. Partial correlation coefficients were computed by controlling the effect of the remaining three variables (components or expectation) (Table 3 and Figure 6). Expectation scores in REAL were positively correlated with SA1 (ρ = 0.67, *p* = 0.02), while they were inversely correlated with CA1 (ρ = −0.82, *p* < 0.01).

**TABLE 2:** Partial correlation coefficients of pairs between SA and CA components for REAL.

	SA1	SA2	CA1	CA2
SA1	-	ρ = −0.23 *p* = 0.54	ρ = 0.69 *p* < 0.01	ρ = −0.20 *p* = 0.50
SA2	-	-	ρ = 0.58 *p* = 0.04	ρ = 0.19 *p* = 0.53
CA1	-	-	-	ρ = 0.35 *p* = 0.24

SA1 and SA2 for somatosensory afferent associating components; CA1 and CA2 for cognitive/affective associating components.

**TABLE 3:** Partial correlation coefficients of pairs between SA/CA components and expectation scores for REAL.

	SA1	SA2	CA1	CA2	Expectation
SA1	-	ρ = −0.51 *p* = 0.09	ρ = 0.84 *p* < 0.01	ρ = −0.30 *p* = 0.34	ρ = 0.67 *p* = 0.02
SA2	-	-	ρ = 0.73 *p* < 0.01	ρ = 0.03 *p* = 0.93	ρ = 0.56 *p* = 0.06
CA1	-	-	*-*	ρ = 0.38 *p* = 0.22	ρ = −0.82 *p* < 0.01
CA2	-	-	-	*-*	ρ = 0.23 *p* = 0.47

SA1 and SA2 for somatosensory afferent associating components; CA1 and CA2 for cognitive/affective associating components.

On the other hand, CA1 in PHNT was positively correlated with the expectation scores (ρ = 0.57, *p* = 0.03). CA1 showed marginal positive correlation with the expectation scores (*r* = 0.50, *p* = 0.06).

## 4. Discussion

Acupuncture is a clinical intervention that consists of multiple therapeutic components of specific effects from needling insertion (i.e., somatosensory afferent) and non-specific effect from patient’s belief and knowledge in acupuncture treatment (i.e., cognitive/affective) (Ho et al., 2021). Despite accumulating clinical evidence of acupuncture stimulation/treatment in patients with various diseases and even in healthy subjects, the underlying mechanism that explains the specific and non-specific effects of acupuncture has not yet been fully investigated. In this study, we attempted to identify specific underlying brain mechanisms for these two therapeutic components induced by compound stimulus of somatosensory stimulation and therapeutic contextual manipulation, which is known to be essential in a typical clinical setting. Using our team’s novel experimental setting, we were able to separate two therapeutic components during the fMRI scan.

However, each participant’s brain activation was not simply manipulated by the experimental protocols as we intended. Not only the manipulated therapeutic components (i.e., somatosensory afferent and cognitive/affective) but also non-therapeutic components, which were implicitly engaged with the protocols, and potential therapeutic components, which could exist in both REAL and PHNT, might interact with each other. Thus, model-based analysis (e.g., voxel-wise GLM) had difficulties assessing these underlying brain processes.

To address the limitation of voxel-wise GLM, we performed data-driven source separation for brain responses by REAL and PHNT. Concatenated BOLD data were decomposed into statistically independent components (ICs), and the dependencies of these ICs on the protocols were assessed (IC-wise GLM). Thus, the combination of experimental dissociation (i.e., REAL and PHNT) and analytic dissociation (i.e., IC-wise GLM) could identify brain response patterns in aspects of somatosensory afferent and cognitive/affective process components.

The temporal concatenated ICA for IC-wise GLM is based on the heterogeneity of brain response patterns across repeated stimulations. REAL and PHNT induced activation in multiple brain regions, which was demonstrated in group average maps from voxel-wise GLM. However, brain activation also had intrinsic variabilities across repeated stimulations during scans, which were variances or standard errors of PEs in the voxel-wise GLM. For instance, a previous fMRI study showed that repeated needling stimulation induced HR decreases as a group average; however, responses of HR increases (Napadow et al., 2013) was also shown in minority in stimulations. This increased HR was accompanied by a different pattern of brain response from that of decreased HR stimulations (Napadow et al., 2013). In addition, habituation from repeated stimulations could decrease voxel activation differentially according to brain regions (Klingner et al., 2011), which causes altered brain response patterns for the same stimulation. Moreover, brain response patterns for somatosensory process could differ across individuals due to behavioral characteristics (Chae et al., 2014). We concatenated REAL and PHNT for IC-wise GLM, which could enhance variability for somatosensory afferent and cognitive/affective process in addition to the heterogeneity in brain responses to repeated stimulations.

Then, we performed GLM analysis for the time series of each IC, which was comparable with voxel-wise GLM that used time series of voxels as independent variables. Instead of FWE correction for whole brain voxels in voxel-wise GLM, our IC-wise GLM performed a Bonferroni correction for multiple comparisons of the resulting 39 ICs, which carried out REAL and PHNT-associated ICs (i.e., SA1, SA2, CA1, and CA2 for REAL; and CA1 and CA2 for PHNT). Moreover, paired comparisons between REAL and PHNT showed that REAL had statistically stronger SA1 and SA2 associations than PHNT. Thus, REAL-specific SA1 and SA2 were identified as somatosensory afferent components according to the experimentally designed dissociation. CA1 and CA2 were identified as cognitive/affective process components because they are common to both REAL and PHNT. Furthermore, the component separations within somatosensory or afferent cognitive/affective process indicate the significance of intrinsic variabilities in brain response patterns.

The thresholded spatial maps of SA1 include SMA, bilateral aIns, and PAG, which are involved in interoceptive attention and motor reaction processes. aIns plays a crucial role in the salience network in the detection of external salient stimuli (Menon and Uddin, 2010) and interoception (Craig, 2009). In particular, activation of the left aIns was found for positive and affiliative emotional feelings from parasympathetic afferents, whereas activation in the right aIns was shown for arousing to the body from sympathetic afferents (Craig, 2002, 2009). PAG is involved in descending control circuitries for somatosensory (Goksan et al., 2018) and motor (Koutsikou et al., 2017) processes to react against external stimuli. SMA is known to be crucial for motor behavior process for action preparation and initiation (Nachev et al., 2008), which also shows activation by multiple sensory modalities (Korvenoja et al., 1999; Lanzilotto et al., 2016; Lima et al., 2016).

While, SA2 includes brain areas of left (ipsilateral) SII and pIns, which are associated with the somatosensory representation process. Indeed, SA2 response was positively correlated with somatosensory sensations in REAL. Studies with somatosensory stimulation have shown ipsilateral activation of SII for tactile representation and perception process (Yu et al., 2017; Lamp et al., 2018; Pala and Stanley, 2022). Moreover, ipsilateral SII activation was accompanied by distinct relationships from contralateral SII regarding functional connectivity (Yu et al., 2017) and descending connectivity modulation (Fardo et al., 2017), supporting the lateralized SII contribution to SA2. pIns is also involved in somatosensory processes (Yeo et al., 2011; Rachidi et al., 2021). Studies have shown nociceptive-specific pIns activation by somatosensory afferent (Craig et al., 2000; Mazzola et al., 2012), suggesting the role of pIns in somatosensory perception (Oertel et al., 2012). Moreover, our previous study demonstrated that REAL inhibited pIns functional connectivity with default mode network and reduced pain level of low back pain patients (Lee et al., 2019).

Moreover, we found that the cardiac response was associated with the decoupling of somatosensory afferent process. Strong coupling between SA1 and SA2 was associated with a greater decrease in HR. Studies with acupuncture stimulation showed parasympathetic cardiac response during stimulations (Nishijo et al., 1997; Uchida et al., 2018; Nakahara et al., 2021), which could be induced by neural circuitry under the brain stem (Guyenet, 2006; Liu et al., 2020). Neuroimaging studies have reported cardiac associations with brain regions of SA1 and SA2, including aIns (Guo et al., 2016), PAG, and pIns (Napadow et al., 2008; Beissner et al., 2013), which suggests that somatosensory perception and reaction are related to the parasympathetic cardiac response. However, causal relationship between SA1/SA2 and the cardiac response was not clearly elucidated in this study.

Again, the thresholded spatial map of CA2 includes V5/MT+, SI, SII, PL, and PCC for bilateral hemispheres, which are involved in dorsal attention network and somatosensory associating processes. V5/MT+ is essential for visual motion perception (Newsome and Paré, 1988; Rees et al., 2000). SI and SII play roles in somatosensory representations for coding and perception (Lamp et al., 2018; Romo and Rossi-Pool, 2020). Studies have shown activation in SI and SII in visual-induced vicarious sensation (Keysers et al., 2010; Schaefer et al., 2012; Bolognini et al., 2014; Makary et al., 2018), which is accompanied by body ownership (Botvinick and Cohen, 1998) or social mirror mechanisms (Keysers et al., 2010). Moreover, brain spatial parcellation with 7 network estimations showed that CA2 included dorsal attention network for PL and PCC, supported CA2 association with vicarious sensation (Vossel et al., 2014; Machner et al., 2021).

CA1 includes dlPFC, IFG, MCC, SII, insular cortex, and supramarginal gyrus, which involves multiple domains of brain function with fronto-pariatal network. The spatial parcellation map using the 7-network demonstrated that IFG, supramarginal gyrus, and insular cortex corresponded to ventral attention network, which is implicated in stimulus-driven attentional control (e.g., reorienting and filtering) (Vossel et al., 2014). MCC corresponds to dorsal attention network, which is involved in top-down orienting of attention (Vossel et al., 2014). Moreover, MCC showed activation with pain for observing (Zhao et al., 2020; Ardizzi et al., 2021) and perception (Schwedt et al., 2014; Bi et al., 2021), and coupling with somatosensory regions (Oane et al., 2020). dlPFC is crucial for behavioral execution process in fronto-pariatal network (Yamagata et al., 2012; Cieslik et al., 2013; Rozzi and Fogassi, 2017). For instance, placebo mechanism is implicated in the top-down execution of dlPFC (Wager and Atlas, 2015; Schenk and Colloca, 2019). Furthermore, CA1 response to PHNT was positively correlated with the expectation for treatment, which is essential for initiating and maintaining placebo effect (Price et al., 2008). Thus, the spatial map of CA1 indicated that the co-working of multiple domains of brain function led top-down execution.

The coupling of CA1 and CA2 was positively correlated with HR decrease after the 4-s stimulation periods for both REAL and PHNT. Our previous studies with PHNT also showed the delayed HR decrease for 6 s (Lee et al., 2014; Makary et al., 2018), suggesting an effect of the top-down execution process on the delayed HR decrease. Moreover, the coupling between CA1 and CA2 for PHNT showed positive correlation with somatosensory sensation (i.e., vicarious sensation) (Figure 5C). Thus, interaction between fronto-pariatal and dorsal systems may lead to the establishment of a vicarious sensation in PHNT (Corbetta et al., 2008). Furthermore, CA1 showed positive correlation with the expectation (general belief for acupuncture treatment) for PHNT, indicating the establishments of the needling credibility and the vicarious sensation were associated with the expectation for acupuncture.

REAL induced HR decrease during the stimulation and the delayed period. While, PHNT induced HR decreases only for the delayed period, indicating that the contextual manipulation could lead to the delayed HR decreases. Moreover, the delayed HR decreases in REAL and PHNT were associated with CA1, suggesting effects of the top-down execution process on the delayed HR decrease. However, the HR decreases during stimulation in REAL were associated with coupling between somatosensory representative and interoceptive attentional processes. Thus, two different brain mechanisms could affect the heart rate decreases in acupuncture treatment.

Again, we found different temporal characteristics among the components. The peak timing of the optimized HRF indicated that the activation of SA2 and CA1 was more sustained or delayed than that of SA1. Studies have shown peak timing differences of the optimized HRF according to cortical and subcortical regions (Steffener et al., 2010; Lewis et al., 2018; Sclocco et al., 2018). Moreover, the peak latency of BOLD response showed differences between brain regions for somatosensory afferent and affective processes regarding tactile pattern discrimination processes (Hegner et al., 2017), supporting the peak timing delay of CA1 compared with SA1. Furthermore, somatosensory stimulation induced stronger aIns and SMA activation during onset timing than sustained phases, whereas activations in SII and pIns remained during sustained phases (Hu et al., 2015). Thus, BOLD responses for SII and pIns were longer than those for aIns and SMA, supporting the late peak timing of SA2 compared to SA1.

Finally, partial correlation analysis revealed intrinsic interactions of SA and CA components in REAL. The partial correlation network of whole pairs of components demonstrated that SA1 and SA2 were not directly related, however, CA1 linked between SA1 and SA2 (Figure 6). These relationships suggested a bi-directional interaction between cognitive/affective and somatosensory process. While, CA2 for goal-directed attention showed no relationship with other components, indicating that the cognitive-somatosensory interaction in REAL differed from goal-directed vicarious sensation mechanism in PHNT. Moreover, we found direct expectation effects (general belief for acupuncture treatment) on CA1 and SA1 using partial correlation analysis (Figure 6 and Table 3). Higher expectation induced weak CA1 responses but strong SA1 responses, which was distinct from the positive expectation effect on CA1 in PHNT. This specific phenomenon may be associated with a characteristic of acupuncture, which induces interoceptive attention for somatosensory input (Craig, 2009; van Ede et al., 2014; Kuehn et al., 2016).

This study has some limitations. Regarding the experimental aspect, a protocol for cognitive/affective dissociation from acupuncture treatment was not incorporated in this study. Our results with IC-wise GLM showed that the four separated ICs, including CA1 and CA2, for cognitive/affective process; however, we could not assess somatosensory afferent process without cognitive/affective interaction.

In conclusion, we identified the four separated ICs induced by compound stimulus of acupuncture. Brain correlates for somatosensory afference were separated into two ICs for interoceptive attention/motor reaction (SA1) and somatosensory representation (SA2) process. Coupling between SA1 and SA2 was associated with strong HR decrease during stimulation. Brain correlates for cognitive/affective modulation were separated into two ICs for executive control (CA1) and goal-directed sensory process (CA2). CA1 is associated with acupuncture sensation and delayed HR decrease. Moreover, partial correlation network with these components suggested a bi-directional interaction between cognitive/affective and somatosensory afferent process, which differed from vicarious sensation mechanism in PHNT. Furthermore, the expectation for the treatment negatively affected CA1 but positively affected SA1 in REAL, indicating a specific bottom-up process associating interoceptive attention for acupuncture treatment. Our findings on brain interactions in acupuncture treatment elucidated the underlying mechanisms of interaction between somatosensory afference and therapeutic contextual manipulation, which might be a specific response for acupuncture.

## Data availability statement

The data supporting the conclusions of this article will be made available by the authors, without undue reservation.

## Ethics statement

The studies involving human participants were reviewed and approved by KHNMC-OH-IRB 2009-006. The participants provided their written informed consent to participate in this study.

## Author contributions

KP conceptualized the study design. CJ, JK, and KP collected the data. CJ and KP analyzed and interpreted the data. CJ wrote the first draft of the manuscript. All authors contributed to critically revising the manuscript and approved the final manuscript.

## Supplementary material

Supplementary Figure 1 REAL led to somatosensory afferent by conducting needling stimulation in addition to contextual manipulations with visual information in stimulation session. For REAL, MRI-compatible needle (0.3 mm x 30 mm, titanium needle, DongBang Co., Korea) was inserted into left ST36 (Zusanli) before the scan started. PHNT led to only contextual manipulations and needling credibility without somatosensory afferent by displaying video for needling stimulation with verbal instruction inducing a context of acupuncture treatment in preparatory session.
