# Supplementary figures and images for: Cognitive and affective interaction with somatosensory afference in acupuncture–a specific brain response to compound stimulus
Source: Front Hum Neurosci. 2023 Jun 21;17:1105703. doi: 10.3389/fnhum.2023.1105703 (PMC10321409; doi:10.3389/fnhum.2023.1105703)

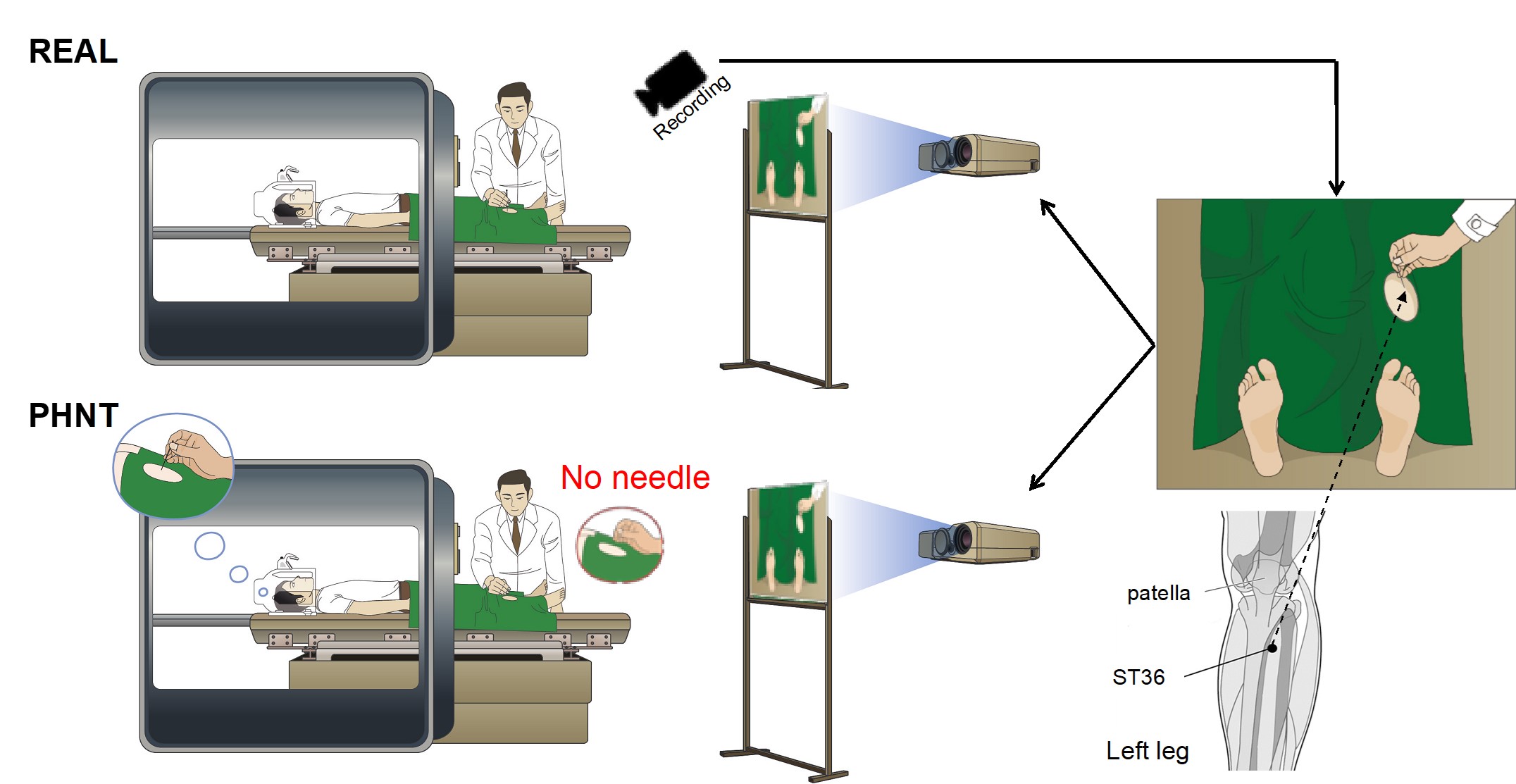

Supplement: Supplementary Figure 1 — REAL led to somatosensory afferent by conducting needling stimulation in addition to contextual manipulations with visual information in stimulation session. For REAL, MRI-compatible needle (0.3 mm x 30 mm, titanium needle, DongBang Co., Korea) was inserted into left ST36 (Zusanli) before the scan started. PHNT led to only contextual manipulations and needling credibility without somatosensory afferent by displaying video for needling stimulation with verbal instruction inducing a context of acupuncture treatment in preparatory session. [file Image_1.jpg]
